# Cross section measurements of ^151^Eu(^3^He,5n) reaction: new opportunities for medical alpha emitter ^149^Tb production

**DOI:** 10.1038/s41598-020-57436-6

**Published:** 2020-01-16

**Authors:** A. N. Moiseeva, R. A. Aliev, V. N. Unezhev, V. A. Zagryadskiy, S. T. Latushkin, N. V. Aksenov, N. S. Gustova, M. G. Voronuk, G. Ya. Starodub, A. A. Ogloblin

**Affiliations:** 1 National Research Center “Kurchatov Institute”, 1, Akademika Kurchatova pl., 123182 Moscow, Russia; 2 Chemistry Department, Moscow State University, 119991 Moscow, Russia; 3 Flerov Laboratory of Nuclear Reactions, Joint Institute for Nuclear Research, Joliot-Curie 6, 141980 Dubna, Russia

**Keywords:** Nuclear chemistry, Experimental nuclear physics

## Abstract

Method for production of alpha emitter ^149^Tb by irradiation of ^151^Eu with 70 MeV ^3^He nuclei is proposed. For the first time, the cross sections for the formation of isotopes ^149,150,151,152^Tb were measured experimentally using a stack foil technique in the ^3^He particles energy range 70 → 12 MeV. The thick target yield of ^149^Tb is 39 MBq/μAh, or 230 MBq/μA ^149^Tb at saturation. The optimal energy range from the point of view of radioisotopic purity is 70 → 40 MeV. At these conditions about 150 MBq/μA ^149^Tb can be produced in 8 hours irradiation, which is sufficient for therapeutic applications. The main impurities are ^150^Tb (~100% in activity) and ^151^Tb (~30% in activity). The proposed method surpasses its counterparts by the high content of the target isotope in the natural mixture and the simplicity of the radiochemical separation of ^149^Tb from the bulk target material.

## Introduction

Targeted alpha-radiotherapy is considered one of the most promising ways for cancer treatment^1–3^. Alpha particles have 2-3 orders of magnitude greater linear energy transfer (LET) than beta particles^1^, therefore the application of alpha emitters allows to target small objects such as micrometastases and single cancer cells more effectively as compared to beta-emitting radionuclides used today. For this purpose, a number of radiopharmaceuticals are considered^4^, in which molecules with a small weight (^225^Ac-PSMA-617)^5^, peptides (^213^Bi-DOTATOC)^6^, or antibodies (^213^Bi-HuM195mAb)^7^ are used as a vector. However, the use of targeted alpha radiotherapy is limited primarily by the limited availability of suitable alpha emitters. Radionuclide ^149^Tb is considered one of the few candidates for targeted alpha-radiotherapy^8^. It has a half-life of 4.12 hours and emits alpha particles with an energy of 3.97 MeV (17%). It also decays by electron capture (76%) and positron emission (7%) Unlike ^225^Ac, ^149^Tb does not have daughter alpha emitters in the decay chain, which means that the recoil effect during radioactive decay should not lead to excessive dose burden. The methods for radiolabeling of biomolecules by using bifunctional chelators based on DOTA, DTPA and others have been well developed for rare earth elements (REE). This is a clear advantage of ^149^Tb over ^211^At and ^223^Ra.

^149^Tb is one of four medically relevant terbium isotopes^8^. The beta emitter ^161^Tb is considered therapeutic radionuclide that exceeds ^177^Lu^9^ in its nuclear properties and ^152^Tb can be used for PET. ^155^Tb is suitable for molecular imaging and Auger therapy^10^. The availability of several isotopes with various nuclear properties allows using isotope pairs for theranostics thus enhancing the perspectives of the medical application of the isotopes of terbium. ^149^Tb can also be considered a theranostic radionuclide. Its positron radiation allows visualizing the distribution of the radiopharmaceutical using PET^11^. *In vivo* experiments^12^ showed that ^149^Tb-rituximab can effectively kill single lymphoma cells. The efficacy of ^149^Tb-DOTA-folate conjugate against carcinoma has also been shown in animal studies^13^.

Various reactions have been proposed for the production of ^149^Tb, in particular, under the action of protons^14^ and heavy ions^15–17^ (Table 1); a review can be found in^18–20^. However, the production of this radionuclide is associated with serious difficulties. In the preclinical studies mentioned above, ^149^Tb was obtained in the spallation reaction by irradiating the tantalum target with a proton beam of 1.0–1.4 GeV energy and online mass-separation of isotopes in the ISOLDE (CERN) facility. As a result, 25 MBq of radionuclide had been obtained at the time of radiolabeling. It was proposed^21^ to obtain ^149^Tb by irradiation of ^151^Eu targets with ^3^He nuclei and the thick target yields in the energy range 70 → 40 MeV were experimentally determined. Preliminary results showed that ^149^Tb yields can be high enough to produce therapeutic amounts of a radionuclide. This work is a further study of ^3^He induced reactions on ^151^Eu and the first experimental measurement of their cross sections.

**Table 1:** Main routes of ^149^Tb production.

Reaction	Projectile energy, MeV	Yield, MBq/μAh	Reference
^152^Gd(p,4n)^149^Tb	70 → 30	2600	^14^
^151^Eu(^3^He,5n)^149^Tb	70 → 40	19.4 (for Eu_2_O_3_)	^21^
^142^Nd(^12^C,5n)^149^Dy → ^149^Tb	108	3.3	^15^
^141^Pr(^12^C,4n)^149^Tb	71.5	0.086	^16^
^nat^Ta(p,x)^149^Tb	1000–1400	~3000 (100 g/cm^2^ target)	^20^

## Results

**Figure 1:**
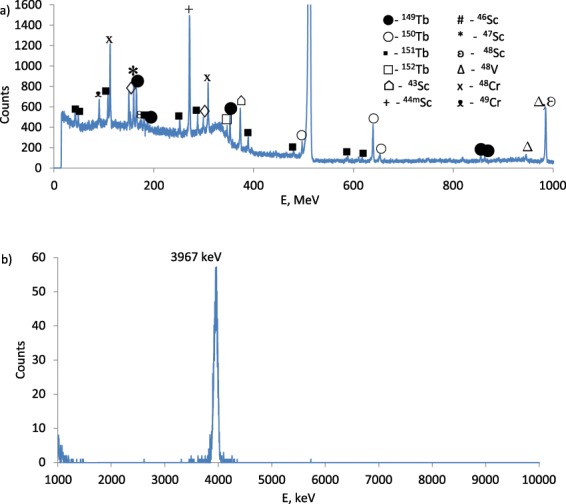
Typical gamma-ray spectrum (**a**) and alpha particle spectrum (**b**) of ^151^Eu target on Ti support irradiated by ^3^He nuclei with incident energy ~50 MeV, measured at ~11 cm distance during 10 minutes 5 h after the end of bombardment (EOB) for (**a**) and measured at ~2 cm distance during 2 minutes 5 h after the EOB for (**b**).

The radioactive isotopes of terbium and gadolinium are formed in the irradiation of a stack of thin (100 μg/cm^2^) ^151^Eu targets by ^3^He nuclei with incoming energy of 70 ± 1 MeV. ^147, 148, 149, 150, 151^Tb and ^147, 149^Gd were identified (Table 2) in gamma-ray spectra (Fig. 1a) of irradiated targets. The alpha activity of irradiated targets was due to ^149^Tb (Fig. 1b) and to a small extent to ^151^Tb. It is not possible to see ^151^Tb peak due to low alpha decay branching (9.5∙10^−3^%), for more spectral data see Supplementary Information.

**Table 2:** Activation products identified in irradiated targets.

Nuclide	Half life	Principal contributing reaction	Q-value MeV	Decay mode	E_γ_, keV	Iγ, %
^147^Tb	1.7 h	^151^Eu(^3^He,7n)^147^Tb	−45.48	EC (100%)	694.4 keV	43.0
^148^Tb	60 m	^151^Eu(^3^He,6n)^148^Tb	−37.62	EC (100%)	784.4 keV	84.4
^149^Tb	4.118 h	^151^Eu(^3^He,5n)^149^Tb	−28.59	EC (83.3%) α (16.7%)	352.2 keV	29.43
^150^Tb	3.48 h	^151^Eu(^3^He,4n)^150^Tb	−20.90	EC (100%) α (<0.05%)	638.1 keV	72.0
^151^Tb	17.609 h	^151^Eu(^3^He,3n)^151^Tb	−12.31	EC (100%) α (0.0095%)	108.1 keV 251.9 keV 287.4 keV	24.3 26.3 28.3
^152^Tb	17.5 h	^151^Eu(^3^He,2n)^152^Tb	−5.15	EC (100%) α (<7E-7%)	344.3 keV	65.0
^147^Gd	38.06 h	^151^Eu(^3^He,p6n)^147^Gd ^147^Tb → ^147^Gd	−40.08	EC (100%)	229.3 keV	63.0
^149^Gd	9.28 d	^151^Eu(^3^He, p4n)^149^Gd ^149^Tb → ^149^Gd	−24.17	EC (100%) α (4.3E-4%)	149.7 keV	48.2

**Figure 2:**
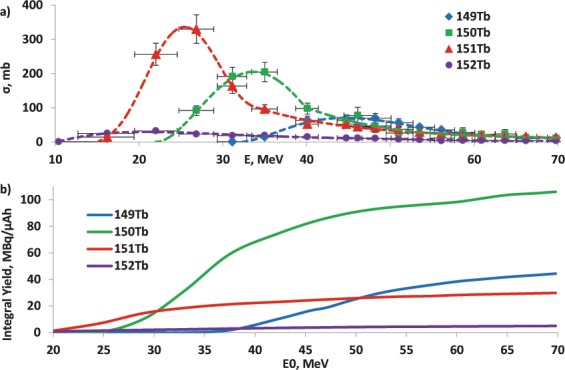
Measured excitation functions for ^151^Eu(^3^He,xn)^154-x^Tb reactions (**a**) and physical thick target yields for reactions ^151^Eu(^3^He,xn)^154-x^Tb (**b**), calculated using measured excitation functions.

The cross sections of nuclear reactions leading to the corresponding terbium isotopes were calculated based on the radioactivity measurements of the irradiated targets. The experimentally obtained excitation functions for the main nuclear reactions are presented in Fig. 2a. By integrating the excitation functions, the physical yields were calculated in the energy range E_0_ → 0, where the incident beam energy E_0_ varied from 70 to a minimum value of ~12 MeV (Fig. 2b).

## Discussion

The physical yield of the ^151^Eu(^3^He,5n) ^149^Tb reaction in the range 70 → 30 MeV was 38.7 ± 7.7 MBq/μAh, that allows one to produce up to 230 MBq/μA on a ^151^Eu metal target up on saturation. In previous work^21^, thick targets from pressed ^151^Eu oxide were irradiated. Our yields based on the experimentally cross sections (Fig. 2a) are in good agreement with obtained in^21^. The saturation yield was 125.0 ± 25.0 MBq/μA for the range 70 → 40 MeV for the target from Eu_2_O_3_^21^ and 161.7 ± 32.3 MBq/μA in this work, recalculated for the same target.

Besides terbium isotopes, peaks of ^147,149^Gd were also detected in the gamma-ray spectra of irradiated targets. They can be formed both by direct reactions and as a result of the decay of ^147^Tb and ^149^Tb, respectively. The relative contribution of these processes to the work was not determined. The ^148^Gd lines were not observed in the spectrum, apparently, due to the low specific activity of this radionuclide.

As follows from the excitation functions shown in Fig. 2a the proposed method does not allow to obtain a product free of radioisotope impurities ^150^Tb and ^151^Tb. They reduce the specific activity of the product and thereby can affect the efficiency of labeling, as well as lead to an undesirable dose burden on the patient. However, both of these impurities have a relatively short half-life. It should also be noted that LET for alpha particles is two orders of magnitude higher than for electrons. Therefore, therapeutic doses of alpha emitters are usually significantly lower than those of the electron emitters. This means that impurity of 150 and 151 isotopes of the same radioactivity will cause a significantly lower damaging biological effect in targeted delivery.

**Figure 3:**
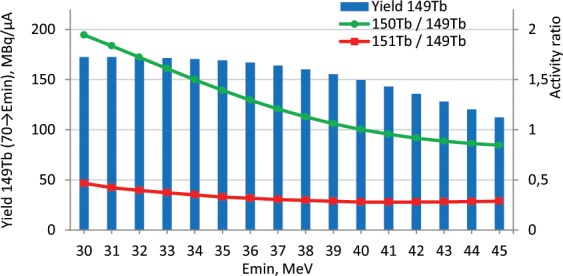
The yield of ^149^Tb and radioisotopic impurities content calculated for 8 h irradiation of metal ^151^Eu target.

In part, the problem of radioisotope impurities can be solved by selecting a suitable range of incident particle energies. The maximum production cross sections for ^149^Tb are ~47 MeV, and for ^150^Tb ~ 35 MeV, ^151^Tb ~ 23 MeV. By changing the lower limit of the energy of the incident particles, one can reduce the amount of impurities, but the yield of the target radionuclide also decreases (Fig. 3). It is proposed to use the range of 70 → 40 MeV as a reasonable compromise between the amount and the purity of the product. Moreover, the activity of the target product ^149^Tb after eight hours will be 150 MBq/µA, which is only 10% less than the maximum possible. At the same time, the content of impurities reduces more than twice in comparison with the range 70 → 30 MeV.

A possible route to obtain ^149^Tb is to use the nuclear reaction ^152^Gd(p,4n)^149^Tb. Its cross section reaches a maximum value of ~250 millibarn at a proton energy of 42 MeV, which inherently provides an opportunity for obtaining of this radionuclide in sufficient quantities for medical use^14^. The yield of this reaction is two orders of magnitude higher than proposed in our work. However, the practical implementation of this path is associated with serious difficulties. The main disadvantage of the proton pathway is the low content of the ^152^Gd target isotope in the natural mixture (0.20%) compared to ^151^Eu (47.8%). Unfortunately, the cross sections for the formation of terbium isotopes with mass numbers of 150 and 151 are not given^14^. Therefore, the question of the content of radioisotope impurities and the optimal range of beam energy remains unsolved. However, it is apparent that in this case, the formation of radioisotope impurities of ^150,151^Tb is inevitable. Another competitive advantage of our method is the further radiochemical processing of the europium target. Europium is one of the few rare earth metals that can be reduced in aqueous solutions to an oxidation state of +2. This makes it possible to quickly and efficiently separate the target radionuclide from the bulk of the target material and traces of gadolinium, and such a technique has already been developed^22^.

Also, the possibility of using ^12^C^15,18^ beams and other heavy ions^23^ to produce ^149^Tb, was repeatedly considered. In this case, both a direct way to obtain by the reaction ^141^Pr(^12^C,4n)^149^Tb is possible, as well as an indirect one: ^142^Nd(^12^C,5n)^149^Dy → ^149^Tb. The latter looks more promising. According to Zaitseva *et al*.^15^, this way can yield tens of GBq for irradiation at a beam current of 50-100 μA. Upon 1.25 hours irradiation of 12 mg/cm^2^ thick natural neodymium target with a beam of ^12^C ions with an energy of 108 MeV and a particle current of 1 μA 2.6 MBq of ^149^Tb were produced. However, no information concerning radioisotopic impurities is given. Irradiation of targets from praseodymium gives a significantly lower activity of ^149^Tb^16^. The weak point of these methods is the lack of accelerators giving intense ^12^C ion beams.

## Methods

### Target preparation and irradiation

**Figure 4:**
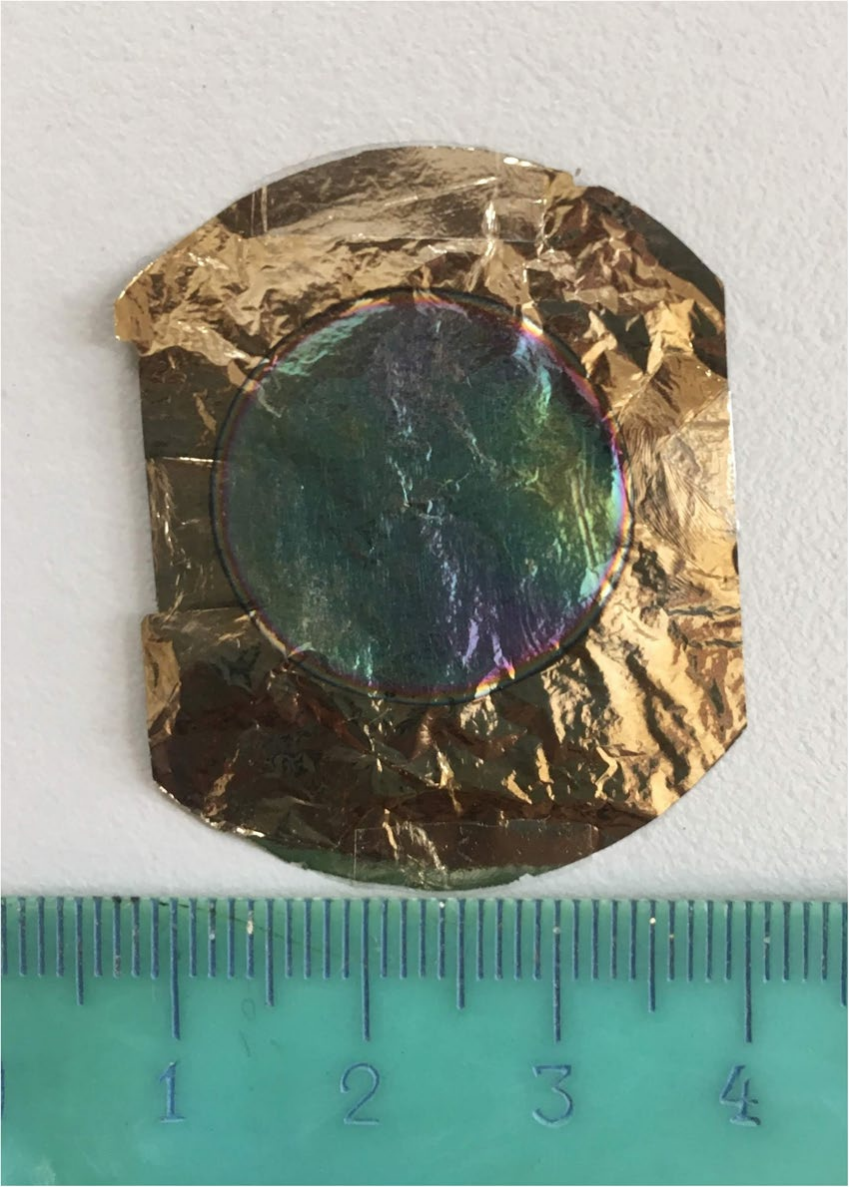
^151^Eu target with a thickness of 100 μg/cm^2^, electrodeposited on a 2 μm Ti support.

^151^Eu of 97.5 ± 0.1% of enrichment in oxide form was used. The material was provided by the National stable isotopes reserve. It contains 0.02% Nd, 0.02% Gd, <0.01% Sm impurities. ^151^Eu targets were prepared by electrodeposition from isopropanol solution on 2 μm thick titanium foil (Fig. 4). Enriched europium oxide powder was dissolved in 4 M nitric acid and evaporated near dryness, then triply evaporated with water, the residue was dissolved in a minimal volume of ethyl alcohol and diluted with isopropanol and placed in an electrolytic cell. Electrodeposition was carried out at a constant current density of 0.058 A/dm^2^ for 2.5 hours at a voltage 100–250 V. The precipitated layer was heated on a hot plate to 400 °C. The thickness of ^151^Eu layer was ~100 μg/cm^2^ that is thin enough to allow the measurement of ^149^Tb alpha particles emitted from the target. The content of Eu on the target was controlled by X-ray fluorescence analysis. To excite X-ray fluorescence, a standard 120 MBq ^109^Cd O-ring source was used. Characteristic x-ray radiation was recorded with Canberra semiconductor Si (Li) detector with an energy resolution of 145 eV for 6.4 keV Fe line. The spectra were analyzed using the WinAxil Canberra software. As a reference 149 μg/cm^2^ europium sample with a diameter of 21 mm was used. It was prepared by evaporation of a standard solution of europium nitrate in isopropanol on a filter paper substrate. Thickness inhomogeneity was controlled using x-ray excitation in the target material through collimator moving along the target.

Up to 10 ^151^Eu/Ti target foils were irradiated in one run using the conventional stack foils technique. Apart from the target foils, the stack also contained 9 μm Al and 13 μm Cu monitors for controlling the beam parameters, and aluminum degraders (53–159 μm), so that the energy ^3^He on each subsequent target was 2–3 MeV less than on the previous one.

The assembled target was irradiated at the U-150 isochronous cyclotron of the National Research Center “Kurchatov Institute”. The initial energy of ^3^He particle beam was 70 ± 1 MeV in the first run and 45 ± 1 MeV in the second. The beam energy was determined by the cyclotron settings and additionally was controlled by the monitoring reactions on Al, Ti, and Cu (see Supplementary Information). The beam current averaged 1 μA irradiation time 1 h. The passed charge was determined by the activation of aluminum monitors, based on the IAEA recommended cross section for ^27^Al(^3^He,x)^24^Na reaction^24^. If necessary, a correction was done in order to take into account the change in the current value during irradiation. In total, two irradiations were performed; the thicknesses of the degraders were selected so that the energy range of ^3^He particles overlaps in both experiments. In Fig. 2a shows the combined result of two irradiations.

### Radioactivity measurements

The radioactivity of the activation products was measured by two methods: α-spectrometry and γ-spectrometry. Gamma-ray spectrometry was carried out on a spectrometer with a high purity germanium detector ORTEC GEM 35P4 Series, the energy resolution of 850 eV for 122 keV line and of 1.5 keV for 1.33 MeV. The measurement was carried out in two geometries at a distance of 11 cm and 6 cm so that the dead time did not exceed 6–7%. Efficiency calibration for the selected measurement conditions was carried out using certified reference point sources ^137^Cs, ^241^Am, ^60^Co, ^152^Eu with an activity of 10 to 100 kBq. Spectra were processed using Spectra Line 1.4.2792 software (LSRM Company, Russia).

Registration of alpha particles was chosen as the main method for the radioactivity measurements for ^149^Tb since the efficiency of registration under the experimental conditions was approximately two orders of magnitude higher than that of gamma rays. Also, the main gamma line of ^149^Tb can interfere with the natural background radiation line 351.9 keV (^214^Pb). Alpha spectrometry was performed without radiochemical separation, registering α particles emitted from the irradiated target surface on an ORTEC Alpha Suite Alpha Duo instrument with ULTRA Ion Implanted Silicon Charged Particle Radiation Detector with an energy resolution of up to 20 keV. The detection efficiency of alpha particles was determined using certified reference sources of ^239^Pu and ^226^Ra.

### Cross-sections calculations and uncertainty budget

The cross sections of nuclear reactions were calculated by activation equation^25^. Nuclear data were used from IAEA database^26^. Energy losses were calculated by SRIM 2008.04 code^27^.

The overall experimental uncertainty was calculated as the square root of the sum of the squares of the individual relative uncertainties. The following uncertainty values of the individual components were taken: detector efficiency - 5–7%, photopeak areas uncertainty varied 1–10% depending on counting statistics, determination of Eu targets thickness and composition - 10%, and nuclear decay data 3%. The overall experimental cross sections uncertainties amounted to about 20%.

Calculating energy uncertainties, it was assumed that the initial beam energy varies within ± 1 MeV (an estimate based on the cyclotron settings). The uncertainty of the thickness of the degraders was estimated at 1 μm by multiple weighing, of copper and titanium foils at 0.1 μm. Based on these data, the possible limits of the energy loss of the beam along the path to each foil were calculated by SRIM 2008.04 code.

## Conclusions

Thus, the use of the ^151^Eu(^3^He,5n)^149^Tb reaction is an effective solution to the problems associated with the production of this very promising radionuclide. In our experiments, low-intensity beams were used, however, up to 20 µA values can be achieved at the Kurchatov Institute isochronous cyclotron U-150. This means that up to 3.0 GBq can be obtained in eight-hour irradiation, which is sufficient for clinical use. Although the therapeutic quantity has yet to be established for ^149^Tb-based preparations, one can estimate from other alpha emitters. For example, for ^213^Bi this value is 10–50 MBq/kg; for ^225^Ac - 20–150 kBq/kg. For ^223^Ra, the recommended dose is 55 kBq/kg. Dosages for ^212^Pb (as [^212^Pb]Pb-TCMC-trastuzumab) are 7.4–21.1 MBq/m^2^, or about 200-500 kBq/kg^28^. It can be assumed that the latter value is closest to therapeutic doses of ^149^Tb. That is, for a single patient, an estimated 10–50 MBq.

The advantage of the method is the availability of target material and the relatively simple radiochemical processing of the irradiated europium. The main drawback is the very limited availability of high-intensity ^3^He beams worldwide.

This method, like other methods not related to electromagnetic mass separation, does not allow one to obtain ^149^Tb free of impurities from short-lived neighboring radioisotopes. The possibility of clinical application of such a product has yet to be proven.

## Supplementary information

Supplementary information

## Data Availability

The datasets generated during and/or analyzed during the current study are available from the corresponding author on request.
