# Supplementary material for: Cross section measurements of 151Eu(3He,5n) reaction: new opportunities for medical alpha emitter 149Tb production
Source: Sci Rep. 2020 Jan 16;10:508. doi: 10.1038/s41598-020-57436-6 (PMC6965643; doi:10.1038/s41598-020-57436-6)
Supplement: Supplementary file 1 — Supplementary information [file 41598_2020_57436_MOESM1_ESM.pdf]

Cross section measurements of ^{151}Eu(^{3}He,5n) reaction: new opportunities for medical alpha emitter ^{149}Tb production

A.N. Moiseeva, R.A. Aliev, V.N. Unezhev, V.A. Zagryadskiy, S.T. Latushkin, N.V. Aksenov, N.S. Gustova, M.G. Voronuk, G.Ya. Starodub, A.A. Ogloblin

Supplementary information

Supplementary Table S1. Measured cross sections for ^{151}Eu(^{3}He,xn)$^{154\text{-}x}$Tb reactions

^{3}He energy, MeV	Reaction Cross-section (mb)			
	^{151}Eu(^{3}He;5n)^{149}Tb	^{151}Eu(^{3}He;4n)^{150}Tb	^{151}Eu(^{3}He;3n)^{151}Tb	^{151}Eu(^{3}He;2n)^{152}Tb
69.8±1.1	12.3±2.5	10.8±7.4	13.0±3.1	3.2±1.0
66.2±1.2	13.1±2.6	10.1±12.1	15.6±5.8	3.5±1.6
63.7±1.3	17.5±3.5	23.6±13.2	15.5±5.2	4.0±1.4
60.9±1.4	18.1±3.6	22.3±10.3	20.5±5.6	4.4±1.4
58.7±1.5	26.2±5.2	27.1±14.5	21.6±5.9	4.0±1.4
56.1±1.5	34.9±7.0	11.6±11.4	25.5±5.7	3.6±1.1
53.5±1.6	43.3±8.7	15.3±15.7	28.8±5.7	6.5±1.4
51.0±1.6	56.2±11.2	26.1±17.2	37.1±8.9	7.7±1.8
48.2±1.8	69.8±14.0	46.9±18.8	42.9±9.2	10.7±2.1
46.1±1.9	46.8±9.4	78.0±23.9	44.3±9.0	11.7±2.2
44.8±1.1	66.1±13.2	61.8±9.8	52.4±7.8	11.5±1.7
40.1±1.4	63.1±12.6	97.9±16.1	52.8±8.1	14.8±2.3
35.0±1.6	15.3±3.1	205.0±28.5	96.7±13.0	19.4±2.8
31.2±1.8	0.4±0.1	191.5±26.9	163.8±21.5	19.0±2.7
26.9±2.1	0.0	91.9±13.7	330.2±42.0	22.9±3.1
22.0±2.6	0.0	0.0	256.3±32.5	32.7±4.3
16.2±3.4	0.0	0.0	14.4±3.1	25.0±3.2
12.0±4.3	0.0	0.0	0.0	0.8±1.2

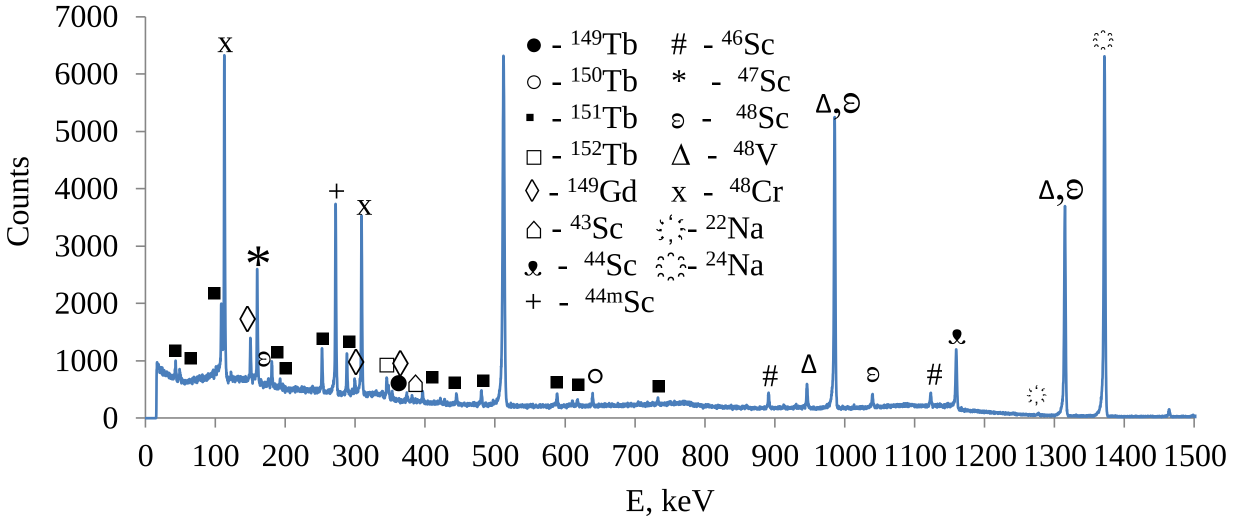

Supplementary Fig. S1. Typical Gamma-ray spectrum of ^{151}Eu target irradiated by ^{3}He nuclei with incident energy ~45 MeV, measured at ~6 cm distance during 1.5 h 5 h after EOB.

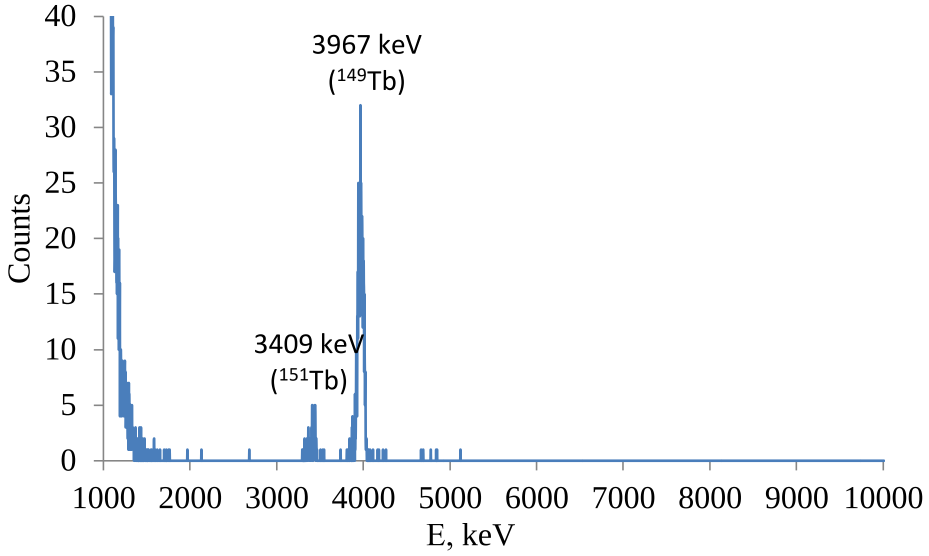

Supplementary Fig. S2. Typical Alpha particle spectrum of ^{151}Eu target irradiated by ^{3}He nuclei with incident energy ~40 MeV, measured at ~2 cm distance during 1.25 h 22 h after EOB.

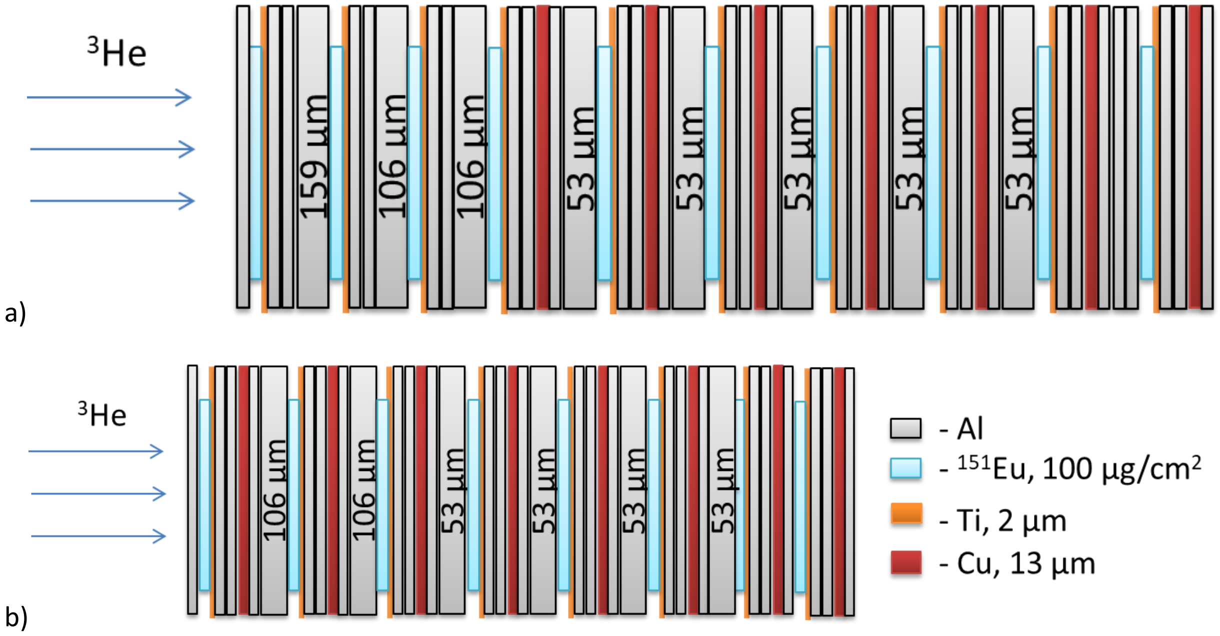

Supplementary Fig. S3. Stack configurations of target foils on the first irradiation (a) and the second (b).

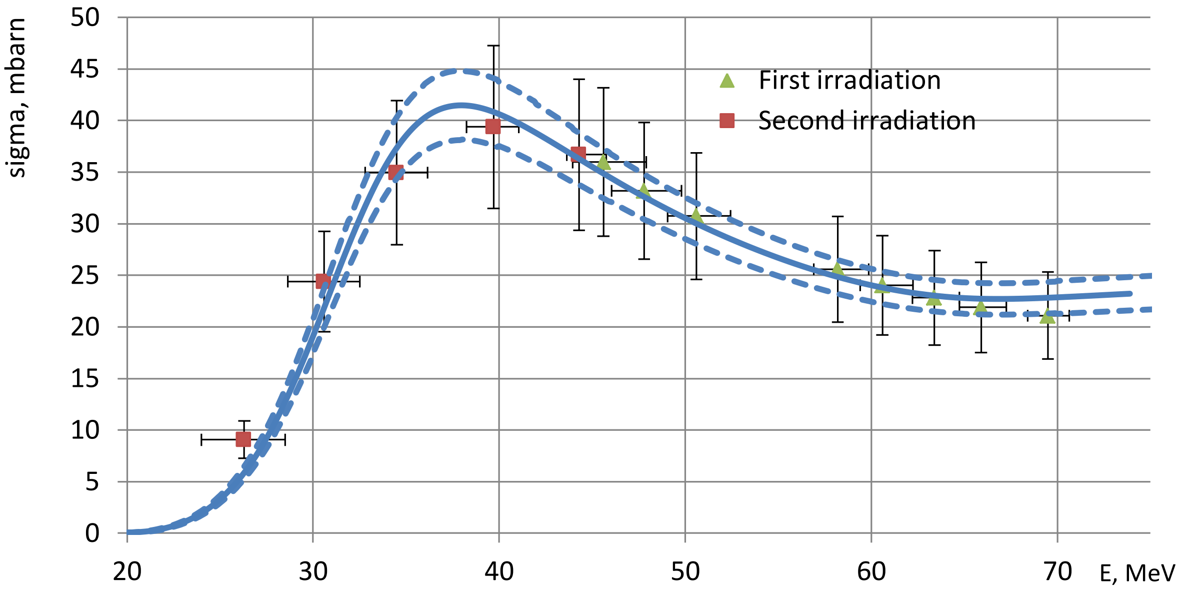
sigma, mbarn
E, MeV
First irradiation
Second irradiation

a)

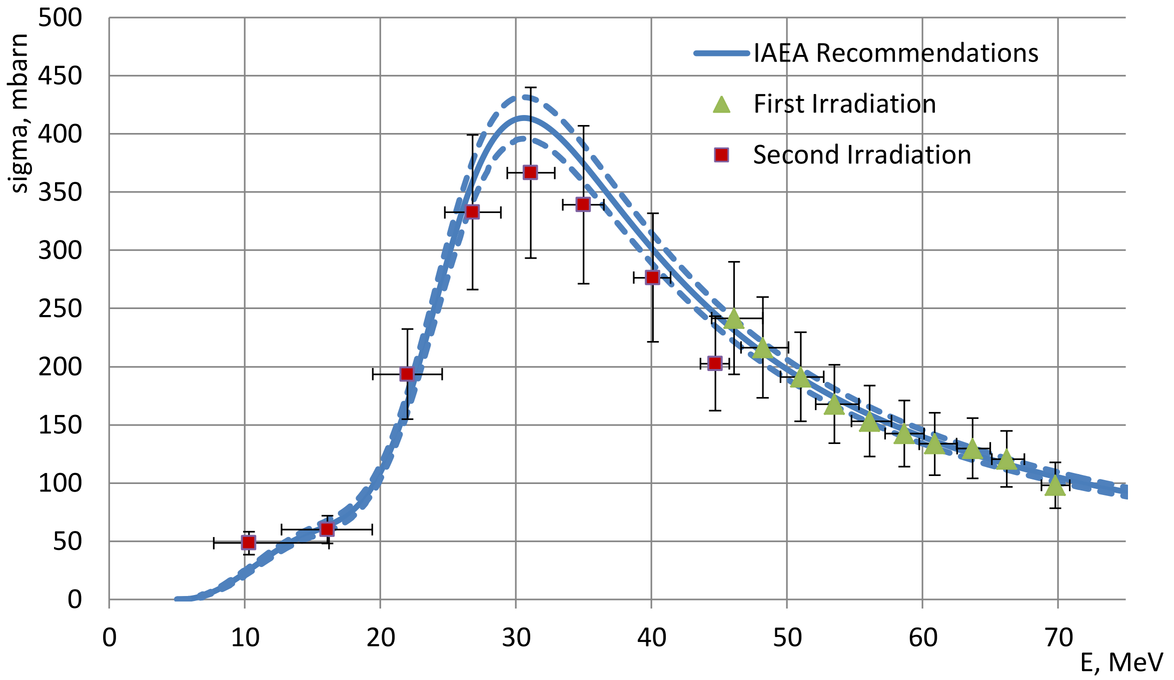
sigma, mbarn
E, MeV
IAEA Recommendations
First Irradiation
Second Irradiation

b)

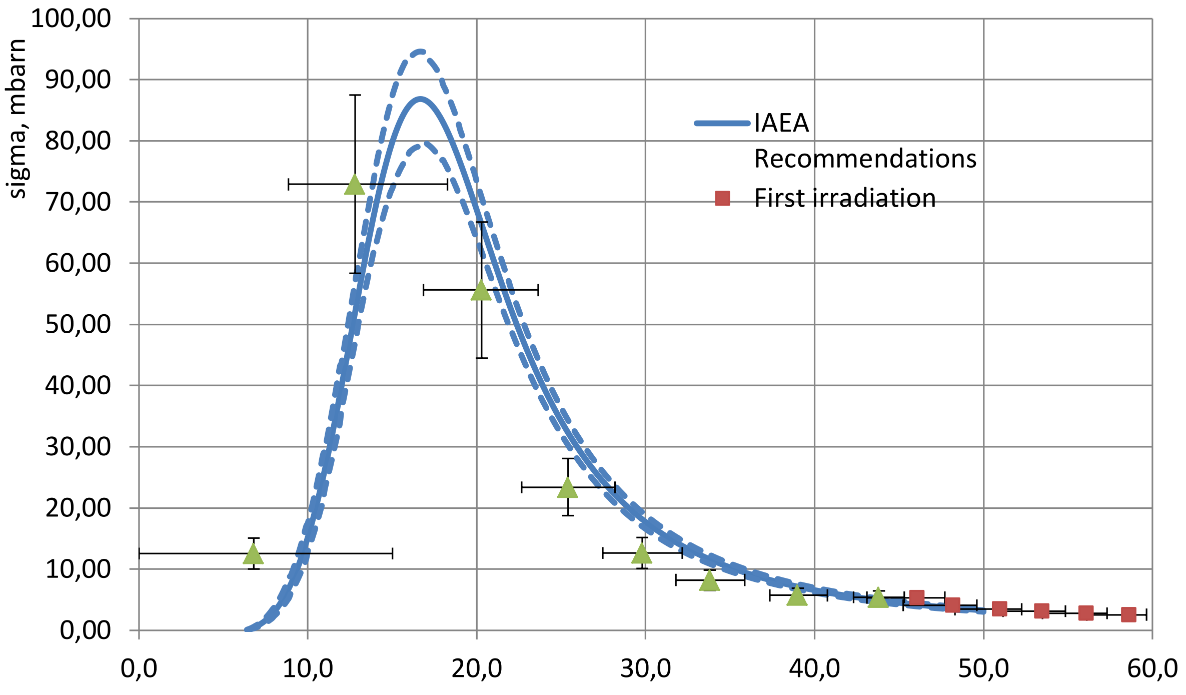

c)

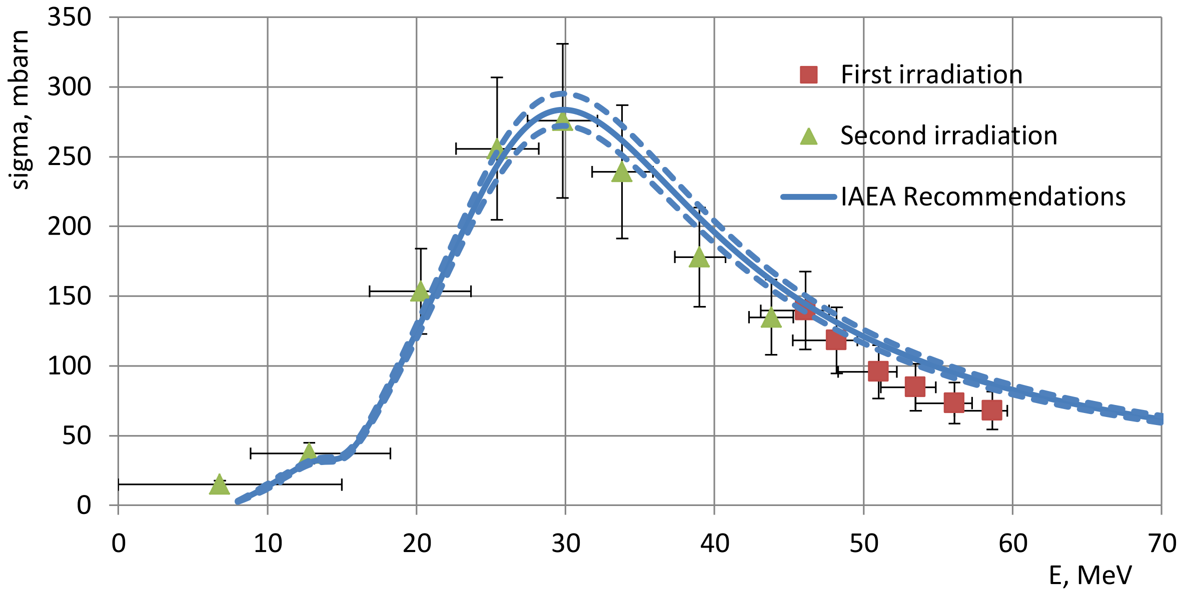

d)

Supplementary fig. S4. Energy control through monitor reactions: a) ^{27}Al(^{3}He,x)^{24}Na; b) ^{nat}Ti(^{3}He,x)^{48}V; c) ^{nat}Cu(^{3}He,x)^{66}Ga; d) ^{nat}Cu(^{3}He,x)^{65}Zn
